# Directional cell movement through tissues is controlled by exosome secretion

**DOI:** 10.1038/ncomms8164

**Published:** 2015-05-13

**Authors:** Bong Hwan Sung, Tatiana Ketova, Daisuke Hoshino, Andries Zijlstra, Alissa M. Weaver

**Affiliations:** 1 Department of Cancer Biology, Vanderbilt University Medical Center, Nashville, Tennessee 37232, USA; 2 Department of Pathology, Microbiology and Immunology, Vanderbilt University Medical Center, Nashville, Tennessee 37232, USA; 3 Division of Cancer Cell Research, Kanagawa Cancer Center, Yokohama 241-8515, Japan; 4 Department of Cell and Developmental Biology, Vanderbilt University Medical Center, Nashville, Tennessee 37232, USA

## Abstract

Directional cell movement through tissues is critical for multiple biological processes and requires maintenance of polarity in the face of complex environmental cues. Here we use intravital imaging to demonstrate that secretion of exosomes from late endosomes is required for directionally persistent and efficient *in vivo* movement of cancer cells. Inhibiting exosome secretion or biogenesis leads to defective tumour cell migration associated with increased formation of unstable protrusions and excessive directional switching. *In vitro r*escue experiments with purified exosomes and matrix coating identify adhesion assembly as a critical exosome function that promotes efficient cell motility. Live-cell imaging reveals that exosome secretion directly precedes and promotes adhesion assembly. Fibronectin is found to be a critical motility-promoting cargo whose sorting into exosomes depends on binding to integrins. We propose that autocrine secretion of exosomes powerfully promotes directionally persistent and effective cell motility by reinforcing otherwise transient polarization states and promoting adhesion assembly.

## Abstract (web summary)

How cells maintain directional polarity when migrating through a complex environment is not well understood. Here Sung *et al.* show that autocrine exosome secretion is required for persistent and efficient *in vivo* cancer cell motility and promotes assembly of adhesion complexes by delivering fibronectin-bound exosomes.

## Graphical abstract

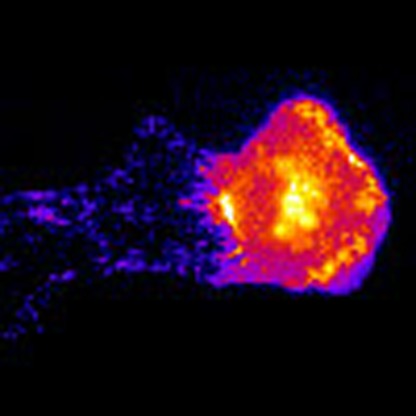

Directional cell movement involves the coordination of multiple subcellular processes. In order for cells to migrate effectively, polarization signals and protrusions must be stabilized to form a dominant leading-edge protrusion^1^. Adhesion assembly is likely to significantly contribute to signal reinforcement at the leading edge^1^; however, the mechanisms by which cells spatially coordinate adhesion assembly during persistent cell migration are poorly understood. In tissues, the extracellular matrix (ECM) environment adds further complexity that could provide conflicting signals to migrating cells if it is not coordinated with chemical gradients.

One potential mechanism that could coordinate initial directional signalling with adhesion formation is vesicle trafficking. Polarization of and trafficking from the Golgi apparatus has been shown to regulate cell migration, although the cargoes are unknown^2^,^3^,^4^. Endocytic recycling of integrins has also been shown to impact cell motility^5^,^6^. Although early studies focused on long- and short-loop integrin-recycling pathways^6^, recent data have identified trafficking of the α5β1 integrin to and/or from late endosomal/lysosomal (LE/Lys) compartments as a key step in cell migration^7^,^8^. In addition, we found that the ECM molecule fibronectin (FN) is endocytosed and resecreted from a LE/Lys compartment to promote *in vitro* migration of fibrosarcoma, breast cancer^9^ and epicardial^10^ cells. Finally, although the cargoes are unknown, additional studies pinpoint secretion from LE/Lys compartments as important for leukocyte chemotaxis and epithelial migration^11^,^12^. Thus, current data suggest that a key decision step in cell migration may be whether otherwise degradatory LE/Lys compartments fuse with the plasma membrane to release important cargo, such as ECM components and their receptors.

Among the components of LE/Lys, compartments that might affect cellular migration are exosomes. Exosomes are small secreted vesicles that carry bioactive cargoes, including growth factors, angiogenic factors, transmembrane receptors, proteinases, ECM molecules and RNAs^13^. It has been shown that purified exosomes can promote adhesion and motility of cells^14^,^15^; however, it is unclear how essential the process of exosome secretion is to cell migration or how it might affect underlying processes such as polarization. It is also unclear how exosome and/or LE/Lys secretion might impact cell migration through complex tissue environments.

To understand how LE/Lys secretion and exosomes control *in vivo* cell motility, we performed xenograft tumour cell motility studies in the chorioallantoic membrane (CAM) of chick embryos. This system is highly advantageous because it enables high-resolution live-imaging studies of cell migration through a physiologic collagen-rich stromal tissue environment^16^,^17^,^18^. We find that exosome secretion is critical for persistent directional migration of tumour cells in the chick CAM, likely due to stabilization of leading-edge protrusions. We further identify exosomes as critical carriers of ECM that promote adhesion assembly, a key step in leading-edge stabilization^19^,^20^,^21^. Targeting of the ECM molecule FN to exosomes depends on specific binding to integrin receptors, ensuring that ECM secreted on exosomes will match cellular receptors.

## Results

### Endolysosomal secretion controls HT1080 migration *in vivo*

**Figure 1:**
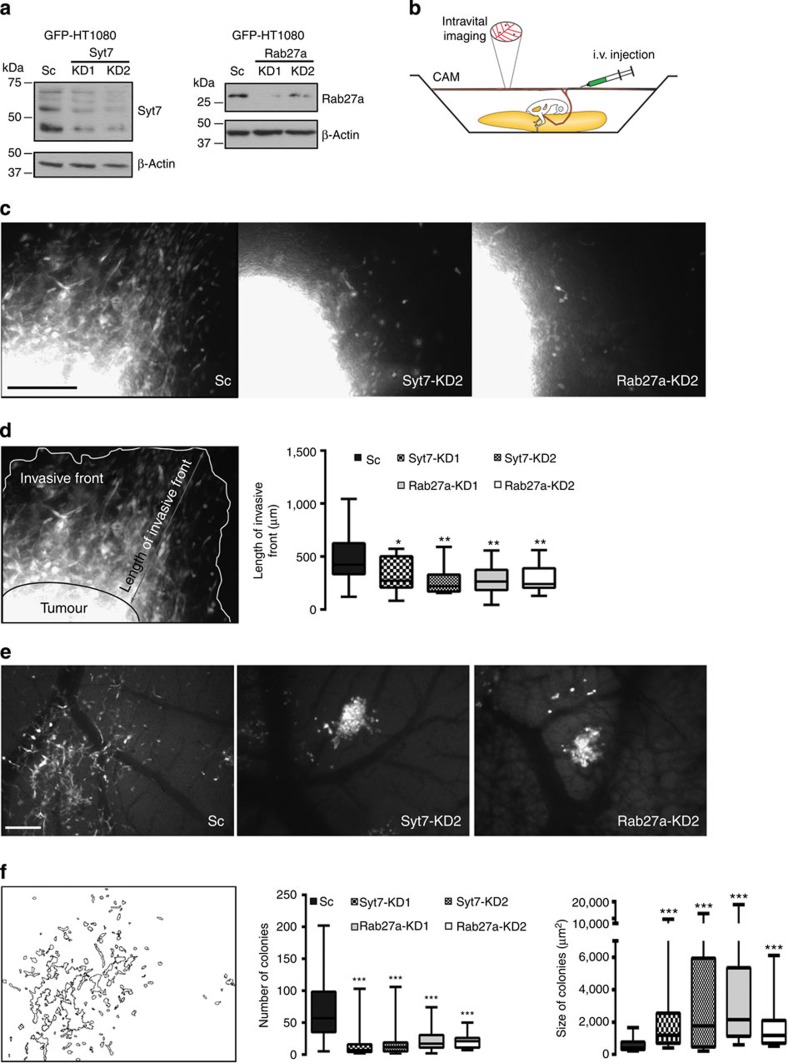
Endolysosomal secretion controls cancer cell motility *in vivo* (**a**) Western blots for the expression of synaptotagmin-7 (Syt7) and Rab27a in GFP-expressing HT1080 cells. Sc, scrambled control; KD, knockdown. (**b**) A schematic cartoon of the chick CAM model. GFP-expressing HT1080 human fibrosarcoma are injected either directly into the CAM or i.v. (**c**) Primary tumours formed on the CAM through subcutaneous injection. Note the lack of cell migration away from Syt7- and Rab27a-KD tumours. Representative images from ≥13 tumours for each condition. (**d**) Quantitation of the length of the migration front, defined as longest distance away from the primary tumour, as shown in image on left. (**e**) Migration away from colonies formed by extravasated cancer cells on the CAM through i.v. injection. Representative images from ≥26 fields. (**f**) Quantitation of colony number and size/image from thresholded images similar to shown example. Each image for KD is representative of two different targeting shRNA-cell lines (KD1, KD2) and all data are from ≥3 independent experiments. Box and whiskers plots are used where the box indicates the 25–75th percentile and whiskers indicate the 5–95th percentile and the black line indicates the median. *P<0.05; **P<0.01; ***P<0.001 compared with Sc using Mann–Whitney *U*-test. Scale bars, 200 μm.

To determine the impact of LE/Lys secretion on *in vivo* motility, we inhibited two canonical regulators of LE/Lys secretion, Rab27a and Synaptotagmin-7 (Syt7), in HT1080 fibrosarcoma cells. Rab27a controls plasma membrane docking of multivesicular late endosomes (MVE)^22^ whereas Syt7 controls fusion of LE/Lys compartments, including MVE, with the plasma membrane^23^,^24^. Syt7 and Rab27a expression were stably downregulated by the expression of specific targeting short hairpin RNAs (shRNAs; Fig. 1a). Green fluorescent protein (GFP)-expressing HT1080 cells carrying the scrambled control (HT1080^Scrambled^) or target-specific shRNA (HT1080^Rab27aKD^ and HT1080^Syt7KD^), were imaged intravitally after local engraftment or intravenous (i.v.) injection into the CAM of chick embryos (Fig. 1b)^17^,^18^. Primary tumours formed after engraftment enable the visualization of cells migrating away from the tumour periphery, while i.v. injection enables high-resolution single-cell tracking of individually extravasated cells. After engraftment, the HT1080^Scrambled^, HT1080^Rab27aKD^ or HT1080^Syt7KD^ cells all formed tumours in the CAM with little obvious difference in tumour size. Examination of the invasive front of each tumour at 4 days after inoculation revealed that fewer KD cells had migrated away from the tumour as compared with control cells (Fig. 1c,d). Following i.v. injection, cells that have extravasated from the bloodstream into the CAM grow to form colonies. Consistent with the previous studies^18^, individual control HT1080 cells had an elongated morphology and migrated rapidly through the CAM to form dispersed, loosely connected colonies (Fig. 1e,f). In contrast, extravasated HT1080^Rab27aKD^ or HT1080^Syt7KD^ cells exhibited a rounded morphology and formed few colonies that were overall larger in size, suggesting ongoing proliferation but defective migration (Fig. 1e,f)^18^,^25^.

### Endolysosomal secretion promotes persistent and fast motility

**Figure 2:**
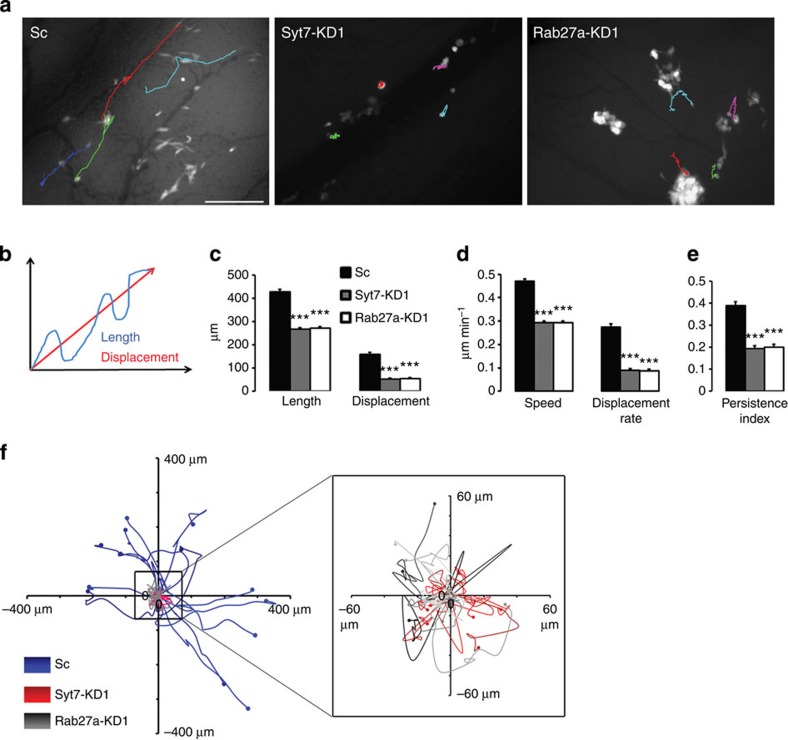
Endolysosomal secretion is critical for persistent and fast migration (**a**) Representative images of HT1080 cells migrating within the CAM. Representative cell tracks are shown. Scale bar, 200 μm. See also Supplementary Movies 1 and 2. (**b**) Depiction of total path length (Length, blue curved line) and productive migration (Displacement, red arrow) calculations from cell tracks. (**c**) Length and displacement plots. (**d**) Speed, calculated by length divided by time. Displacement rate, calculated by displacement divided by time. (**e**) Persistence index, calculated as displacement rate divided by speed. (**f**) Wind–Rose plots from cell tracks. Magnification of boxed area shown on right. Error bars, s.e.m. from four independent experiments (*n*>130 cells for each cell line). ***P<0.001 compared with Sc by Student’s *t*-test.

To directly measure cancer cell motility *in vivo*, we performed time-lapse imaging of cancer cells migrating within the CAM. GFP-expressing HT1080^Scrambled^, HT1080^Rab27aKD^ and HT1080^Syt7KD^ were imaged in the CAM for 16 h during a period of 1–3 days after i.v. injection. Analysis of the movies revealed that HT1080^Scrambled^ exhibited polarized migration, allowing them to move in a directionally persistent manner. Conversely, HT1080^Rab27aKD^ and HT1080^Syt7KD^ cells that initially moved in one direction would frequently stop and reverse course (representative cell tracks shown in Fig. 2a, see also Supplementary Movies 1 and 2), leading to diminished overall displacement from the initial position (Fig. 2b,c). HT1080^Rab27aKD^ and HT1080^Syt7KD^ cells also exhibited an overall defect in the total path length travelled by the cells, regardless of the directionality (Fig. 2b,c). To determine whether there was a distinct defect in directionality in KD cells, separate from any defects in overall speed of movement, the persistence index was calculated by dividing the displacement rate by cell speed (Fig. 2d,e). Fully persistent movement would result in a straight migration track and give a persistence index of 1. Control cells had a persistence index of ∼0.4. By contrast, HT1080^Rab27aKD^ and HT1080^Syt7KD^ cells had persistence indexes of ∼0.2, indicating a strong defect in directionally persistent migration, independent of defects in cell speed (Fig. 2e). Wind–Rose plots of representative cell tracks further illustrate that endolysosomal secretion promotes persistent and efficient *in vivo* migration (Fig. 2f). A similar phenotype was observed in HEp3 squamous carcinoma cells (Supplementary Fig. 1).

### Endolysosomal secretion controls protrusion dynamics

**Figure 3:**
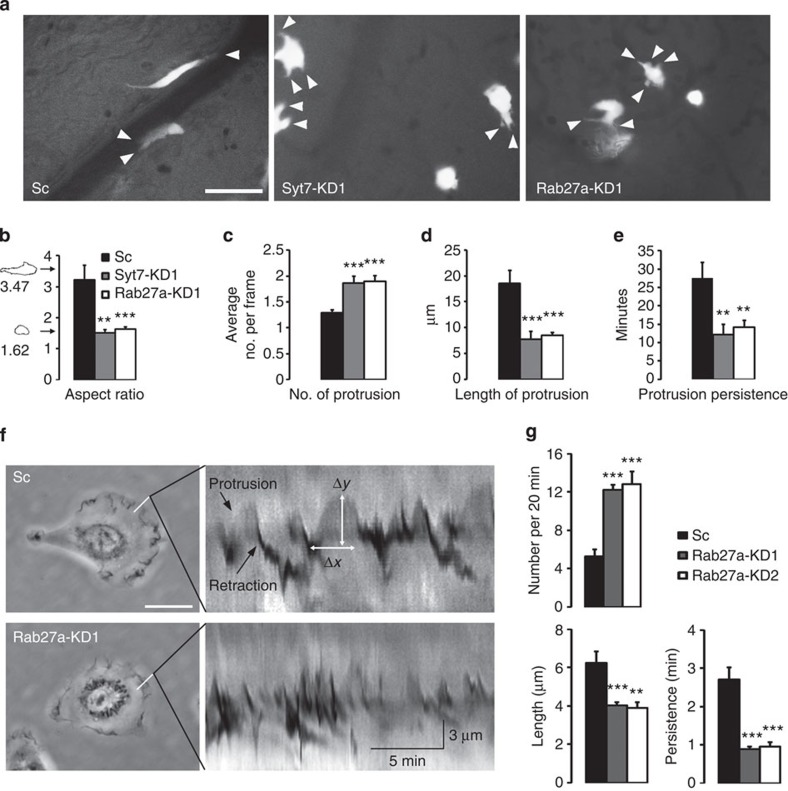
Endolysosomal secretion controls protrusion dynamics *in vivo* and *in vitro* (**a**–**e**) *In vivo* protrusion dynamics of HT1080 cells in the CAM (**a**) Representative images. Arrowheads indicate protrusions. Movies were recorded every 2 min for 60 min. Scale bar, 50 μm. See also Supplementary Movie 3. (**b**) Aspect ratio was calculated as length of major axis divided by length of minor axis of cell. Representative cell morphologies for high (3.47) and low (1.65) aspect ratios are shown at the left of the bar graphs. (**c**) Number of protrusions per cell was counted at each time frame (2 min) and averaged over movies (60 min). (**d**) Length of each protrusion. (**e**) Persistence of each protrusion was measured as the time between appearance and disappearance of each protrusion. *n*>30 cells for each cell line. (**f**,**g**) *In vitro* protrusion dynamics of HT1080 cells on tissue culture-treated dishes. (**f**) Representative kymographs. Note definitions of protrusion persistence (length of time that a protrusion lasts before retraction, Δ*x*) and length of protrusion (Δ*y*). Scale bar on Sc cell image indicates 30 μm. Scale bars on kymograph indicate time and distance axes. (**g**) Quantitation of number of protrusions/time, length of protrusion and protrusion persistence. *n*≥12 cells for each condition. Error bars, s.e.m. from three independent experiments. *P<0.05; **P<0.01; ***P<0.001 compared with Sc by Student’s *t*-test.

To further identify cellular features of migration regulated by endolysosomal secretion, we performed high-magnification live-cell imaging of cells migrating in the CAM. Analysis of the cellular morphology revealed that HT1080^Scrambled^ cells were much more elongated than cells inhibited for LE/Lys secretion (Fig. 3a,b, see also Supplementary Movie 3). It was also apparent that HT1080^Rab27aKD^ and HT1080^Syt7KD^ cells made more numerous but shorter-lived protrusions. Quantitation of the protrusion dynamics revealed that KD cells had a 50% increase in the number of protrusions formed per frame during migration (Fig. 3c), along with a decrease in the length and persistence of protrusions (Fig. 3d,e). These data indicate that LE/Lys secretion strongly contributes to establishment of a persistent leading-edge protrusion *in vivo*, which is critical for maintaining the cell directionality during migration. Interestingly, these results are reminiscent of our previous *in vitro* finding that endolysosomal recycling of ECM promotes the stabilization of leading-edge protrusions^9^. To determine if Rab27a and Syt7 also control the stability of protrusions *in vitro*, we performed live imaging and kymography analysis of the movies. We found a similar *in vitro* phenotype, with HT1080^Rab27aKD^ and HT1080^Syt7KD^ cells making an increased number of short-lived protrusions (Fig. 3f,g). The strong similarity between the *in vitro* and *in vivo* phenotypes suggests that we can exploit powerful available *in vitro* techniques to identify the mechanisms by which endolysosomal secretion controls cell motility.

### Exosomes promote effective cell motility by carrying ECM

**Figure 4:**
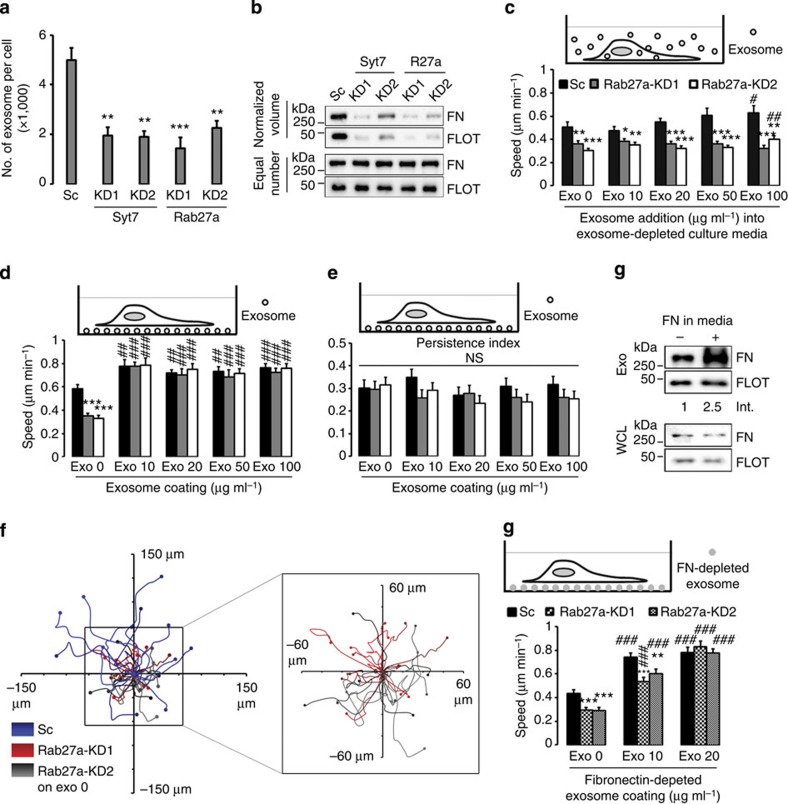
Exosome secretion promotes cell migration. (**a**) Average number of exosomes secreted from endolysosomal secretion-inhibited HT1080 cell lines from *n*≥3 experiments. (**b**) Western blots of exosome cargos. FLOT, flotillin. (**c**) Migration speed of Rab27a-knockdown (KD) HT1080 cells in exosome-depleted media±add-back of purified exosomes (Exo), as indicated on tissue culture-treated plates. (**d**) Speed of Rab27a-KD cells on exosome-coated plates. (**e**) Persistence index calculated from experiments shown in **d**. (**f**) Wind–Rose plots for data shown in **d**, Exo 0. Magnification of boxed area is shown at the right. (**g**) Western blot for FN-depleted exosomes. Numbers below blot represent relative intensity (Int.) of FN (normalized to FLOT) from that blot. WCL, whole-cell lysates; (**h**) Single-cell migration assays on FN-depleted exosome-coated plates. Error bars, s.e.m., from *n*≥29 cells for each cell line from three independent experiments. *P<0.05; **P<0.01; ***P<0.001 compared with Sc by Student *t*-test. *^#^P*<0.05; ^##^*P*<0.01; ^###^*P*<0.001 compared with same cell line on Exo 0 by Student’s *t*-test. NS, not significant.

A specialized endolysosomal compartment that could potentially control cell migration is the MVE, which on fusion with the plasma membrane releases exosome vesicles to the extracellular milieu^13^. To confirm that exosome secretion is inhibited in HT1080^Rab27aKD^ and HT1080^Syt7KD^ cells, exosomes were purified from conditioned media by serial centrifugation and analysed by NanoSight particle analysis and immunoblotting for the exosome marker flotillin and the ECM molecule FN. NanoSight analysis revealed that HT1080^Rab27aKD^ and HT1080^Syt7KD^ cells secreted 2.2- to 3-fold fewer exosomes/cell than control cells (Fig. 4a). Consistent with the reduction in exosome secretion, the immunodetection of flotillin and FN was similarly reduced in exosome preparations from HT1080^Rab27aKD^ and HT1080^Syt7KD^ cells (Fig. 4b, top panel). However, when an equal number of vesicles was loaded on blots, there was no difference in FN or flotillin levels (Fig. 4b, bottom panel).

To determine if autocrine secretion of exosomes was required for *in vitro* cell motility, live imaging of HT1080^Scrambled^ and HT1080^Rab27aKD^ cells was performed in the absence or presence of purified exosomes. In the absence of exogenously provided exosomes, HT1080^Rab27aKD^ cells had a defect in the speed of migration (Fig. 4c,d, ‘Exo 0’ condition). However, unlike the migration *in vivo*, HT1080^Rab27aKD^ did not exhibit diminished persistence compared with HT1080^Scrambled^, as quantitated by the persistence index (compare Fig. 4e, ‘Exo 0’, to *in vivo* migration results in Fig. 2e) and visualized in Wind–Rose plots (Fig. 4f). We speculate that this difference reflects the presence of directional cues *in vivo* that are not present in our *in vitro* motility environment.

Exosomes have the potential to control multiple aspects of cell motility, including stimulation of extracellular receptor signalling and as carriers of ECM to promote adhesion formation^14^,^15^,^26^,^27^. As a first test of these possible roles, exosomes purified from control HT1080 cells were added to the cells in two formats—either added into the media at the time of migration like a growth factor (Fig. 4c) or coated overnight onto tissue culture plates similar to ECM coatings (Fig. 4d). On addition of purified exosomes, HT1080^Scrambled^ cells exhibited increased migration speed, especially when exosomes were coated onto culture dishes. Interestingly, the speed defect of HT1080^Rab27aKD^ cells was fully rescued on exosome-coated plates (Fig. 4d) but not with the addition of exosomes into media containing exosome-depleted serum (Fig. 4c). These data suggest that deposition of exosomes to the substrata where it is accessible to the basal cell surface is important for their ability to promote cell migration.

The presence of FN on exosomes (Fig. 4b), the lamellipodial stability defect of KD cells (Fig. 3) and the selective rescue of Rab27a-KD cells on exosome-coated plates (Fig. 4d) suggest that exosomes may serve a critical function in providing matrix attachment for migrating cells. By immunoprecipitation, FN is on the outside of exosomes, indicating that it is indeed of the expected topology to support adhesion and migration (Supplementary Fig. 2). To determine whether the presence of FN on exosomes is important for their ability to support migration, we prepared FN-depleted exosomes by culturing control cells for 10 days in FN-depleted serum before collecting 48-h conditioned serum-free media for exosome purification. This procedure led to a 2.5-fold decrease in the FN content of exosomes, compared with control preparations (Fig. 4g). When coated onto culture dishes and tested in single-cell motility experiments, FN-depleted exosomes were less efficient at rescuing single-cell motility than control exosomes (compare Fig. 4h to Fig. 4d). Thus, while 10 μg ml^−1^ coating of control exosomes fully rescued motility of Rab27a-KD cells, twice as many FN-depleted exosomes were required to rescue their motility. The rescue of Rab27a-KD cell motility by 20 μg ml^−1^ FN-depleted exosomes could be due to remaining FN on the FN-depleted exosomes or to other exosome cargoes.

**Figure 5:**
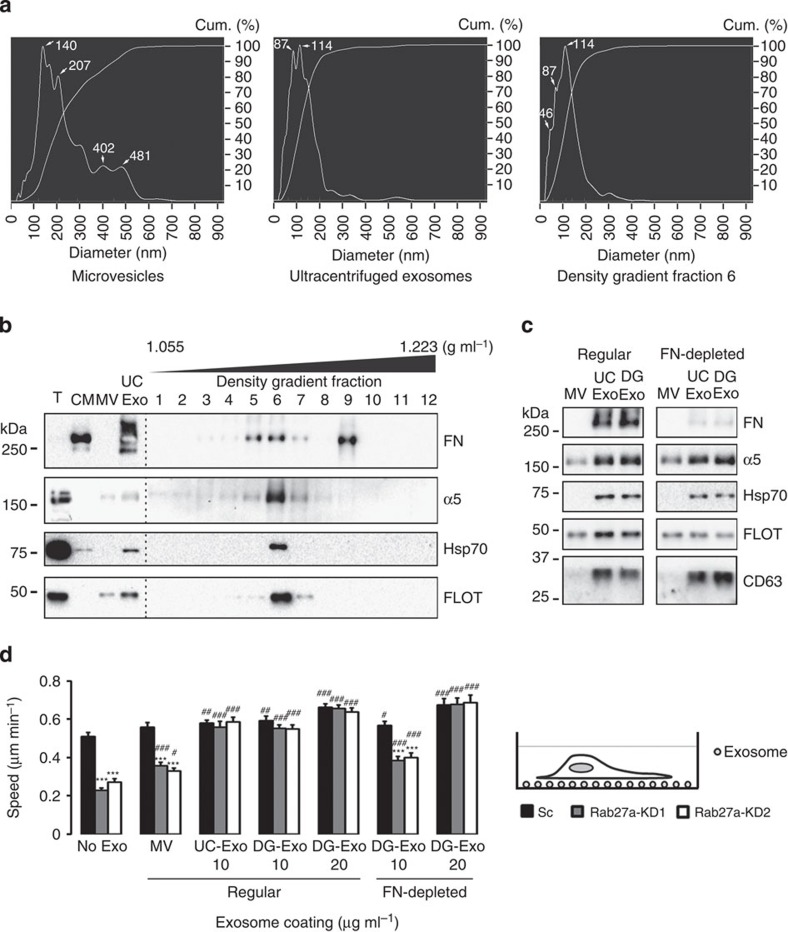
Density gradient-purified exosomes have bound FN and rescue Rab27a-KD cell motility defects Extracellular vesicles were purified by ultracentrifugation. Microvesicle (Microvesicles) and exosome (Ultracentrifuged exosomes) fractions were analysed by NanoSight (**a**). Part of the Ultracentrifuged exosomes fraction was further purified by Optiprep density gradient centrifugation. (**b**) Western blot analysis of the fractions (Density gradient fractions 1–12) for exosome markers HSP70 and flotillin identified Fraction 6 as the major peak containing exosomes. NanoSight analysis of this fraction ((**a**): Density gradient fraction 6) showed a typical exosome size profile similar to the Ultracentrifuged exosomes profile, whereas Microvesicles fraction contained larger vesicles. Both FN and its receptor α5 integrin were present in the Fraction 6 exosome peak. FN was also present separate from exosome markers in Fraction 9. Conditioned media (CM) contained FN but the microvesicle (MV) fraction did not. T=Total cell lysate. Representative blots from *n*=2. (**c**) Western blot analysis of MVs, ultracentrifuge-purified exosomes (UC-Exo) and exosomes present in Fraction 6 density gradients (DG-Exo) from control cells cultured in normal media (Regular) or in FN-depleted media for 10 days. Equal numbers of vesicles were loaded in the gel lanes. Note that FN, HSP70 and CD63 were uniquely present in the exosome samples. Although the lanes are demarcated from each other due to intervening lanes not being shown, the regular and FN-depleted Exo lanes came from the same blot and are directly comparable for all antibodies. (**d**) The ability of various extracellular vesicles to rescue motility of Rab27a-KD cells was tested by coating 10 μg ml^−1^ UC-Exo, microvesicles purified from an equivalent number of cells that produced 10 μg ml^−1^ UC-Exo or 10 or 20 μg ml^−1^ DG-Exo. Similar to results with UC-Exo (Fig. 4d,h), twice as many DG-Exo purified from FN-depleted cells were required to rescue Rab27a-KD speed as Regular DG-Exo. Data from ≥30 cells from ≥3 independent experiments for each condition. Error bars indicate s.e.m. ***P<0.001 compared with Sc by Student’s *t*-test. *^#^P*<0.05; ^##^*P*<0.01; ^###^*P*<0.001 compared with the same cell line on Exo 0 by Student’s *t*-test.

The concomitant decrease in FN and the membrane marker flotillin in exosome preparations from Rab27a- and Syt7-KD cells that were loaded according to equal cell number (Fig. 4b, top blot) suggests that FN is not merely a contaminant of our ultracentrifugation (UC) preparation technique. Furthermore, the size distribution of exosomes and microvesicles in our UC fractions were as expected (Fig. 5a) and approximately threefold fewer MVs are secreted by our cells, suggesting that it is exosomes rather than microvesicles that promote cell motility in our system. However, to test the specificity of exosomes in carrying FN and promoting cell motility, we further purified our UC exosome preparations by sedimentation into a density gradient of Optiprep (DG exosomes). Western blot analysis of the fractions revealed that FN was purified in two peaks—one that included the exosome markers HSP70 and flotillin along with the FN-binding integrin α5 subunit and another peak that did not have any of those markers and probably represents FN fibrils not associated with exosomes (Fig. 5b). Microvesicle preparations contained no FN or HSP70 but did contain a small amount of flotillin and α5 integrin, which are both plasma membrane markers and may be found in both types of vesicles. To determine whether our Optiprep-purified exosomes could rescue cell motility defects of Rab27a-KD cells, we coated plates and performed the same live-imaging assay as we had performed with UC-exosomes. Compared with one-step UC-exosomes from the same conditioned media preparation, Optiprep-double purified exosomes were equipotent in their ability to rescue cell motility (Fig. 5d). By comparison, the microvesicle fraction from the same conditioned media was unable to rescue motility. As with the UC-exosomes (Fig. 4h), FN-depleted DG exosomes were less efficient in rescuing cell motility compared with control DG exosomes (Fig. 5c,d).

These data are consistent with our recent finding that recycling of endocytosed FN from a LE/Lys compartment drives cell motility^9^ and suggest that exosomes are key delivery vehicles for that FN. Consistent with this model, we tested whether endocytosed fluorescent FN was likewise secreted by HT1080 cells migrating in the chick CAM. Indeed, 24 h after i.v. injection of DyLight550-FN-loaded HT1080 cells into the chick CAM, we observed incorporation of the endocytosed FN into streak-like structures reminiscent of adhesions associated with cellular protrusions (Supplementary Fig. 3 and Supplementary Movie 4).

To further verify that the secretion of exosomes rather than non-exosomal LE/Lys cargo is required for motility, we knocked down a component of the endosomal sorting complexes required for transport (ESCRT) machinery that is necessary for intraluminal vesicle formation within MVE^28^. Hepatocyte growth factor-regulated tyrosine kinase substrate (Hrs) is part of the ESCRT-0 complex, and inhibition of Hrs prevents exosome biogenesis (Supplementary Fig. 4, refs 23, 29). As expected, Hrs KD diminished the release of exosomes into conditioned media (Supplementary Fig. 4a–c) and HT1080^HrsKD^ cells had motility defects *in vivo* (Supplementary Fig. 4d,e) and *in vitro* (Supplementary Fig. 4f, Exo 0 and 5g, FN 0 conditions). Consistent with our hypothesis that matrix-containing exosomes promote cell motility, the *in vitro* motility speed defect of HT1080^HrsKD^ cells was fully rescued on culture dishes coated with either exosomes (Supplementary Fig. 4f) or 10 μg ml^−1^ purified FN (Supplementary Fig. 4g).

### Secreted exosomes promote assembly of nascent adhesions

**Figure 6:**
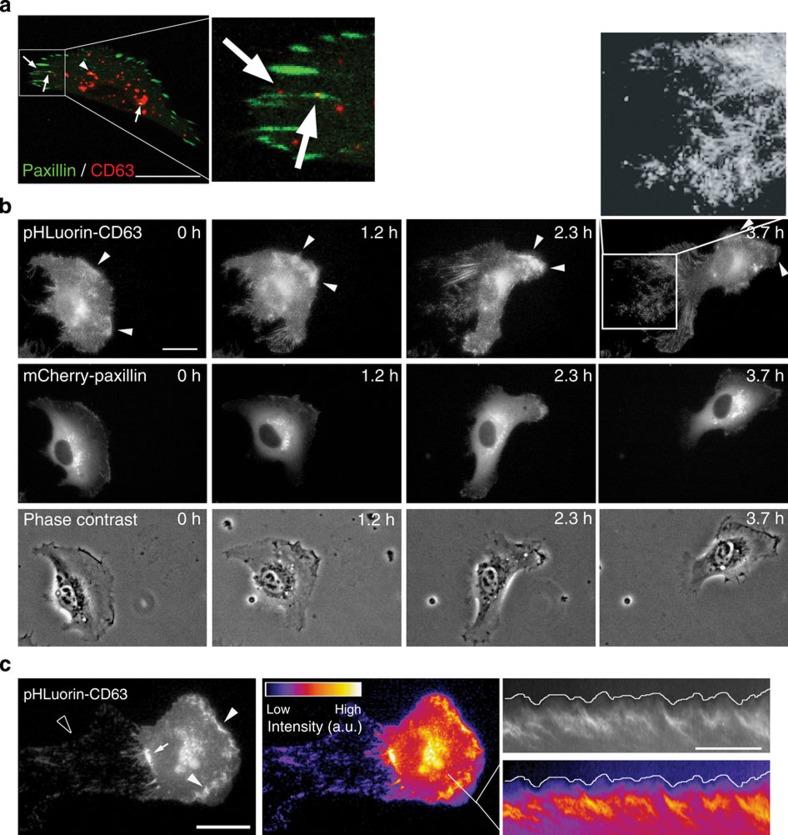
Adhesive exosome trails are left behind migrating cells (**a**) Confocal image of mCherry-CD63 (red) and GFP-paxillin (green)-expressing HT1080 cell from a representative movie from *n*=17 movies on FN-coated (1 μg ml^−1^) MatTek dishes. Arrows indicate co-localization of mCherry-CD63 with adhesions, whereas an arrowhead indicates localization of paxillin within endosomes. Magnification of boxed area is shown right. See also Supplementary Movie 5. (**b**) Image series from a representative epifluorescence movie (1 frame per 2 min, from *n*=10 movies) of a migrating HT1080 cell on FN-coated (1 μg ml^−1^) MatTek dishes transiently transfected with pHLuorin-CD63 to show sites of exosome secretion. Note CD63 fluorescence in ruffles at the front of the cell (arrowheads) and in an adhesive trail left behind the cell. Zoom is brightened and shows adhesive trail. See also Supplementary Movie 6. (**c**) Image from a representative epifluorescence movie with a rapid frame rate (1 frame per 6 s, from *n*=20 movies) to capture the dynamics of pHLuorin-CD63 in protrusions. Filled arrowheads indicate fluorescence in ruffles, whereas open arrowhead points to adhesive trail. The arrow points to rear fluorescence near retracting fibres. Heat map-coloured intensity is shown right. Grayscale and coloured kymographs show protrusion over time across the perpendicular line drawn in heat map image. Note lack of enhanced fluorescence at flat protrusion membrane edge, which is outlined by drawn white lines. See also Supplementary Movie 7. Scale bars, 30 μm.

Our data suggest that a major role of exosomes is to transport ECM and presumably regulate adhesions. To investigate whether exosome-containing MVE are transported to and secreted at specific cellular locations, we performed live confocal imaging of cells expressing the MVE marker mCherry-CD63. To simultaneously visualize adhesions, we expressed GFP-paxillin. As previously reported, CD63-positive tubules and vesicles were observed moving dynamically in cells (Fig. 6a and Supplementary Movie 5). In some cases, these vesicular structures appeared to target peripheral adhesions (Fig. 6a and Supplementary Movie 5, arrows); however, the majority of the CD63 was in endosomes away from the plasma membrane and some CD63-positive endosomes also carried paxillin (Fig. 6a and Supplementary Movie 5, arrowhead).

To definitively identify exosome secretion sites, we constructed a novel imaging probe, which has the pH-sensitive GFP-pHLuorin moiety inserted into the first extracellular loop of CD63 (CD63-pHLuorin). Because pHLuorin-GFP fluorescence is quenched at the acidic late endosomal pH, but fluoresces brightly at the neutral pH of the extracellular environment^30^, it is ideal to track proteins that are exocytosed from endosomes. To validate this construct, we co-expressed it with mCherry-Rab5-Q79L, which both marks and leads to the enlargement of early-to-late-transitioning endosomes^31^, in control and Rab27a-KD cells. Consistent with the construct marking secretory MVE, pHLuorin-CD63 fluorescence was present on the cell surface of control cells, but not of Rab27a-KD cells (Supplementary Fig. 5). In addition, Bafilomycin treatment of Rab27a-KD cells to inhibit acidification of endosomes reveals its presence in internal vesicles. Finally, since many Rab5-positive endosomes will not be fully acidified yet, note the internal co-localization of pHLuorin-CD63 with some Rab5-Q79L endosomes in control cells but less co-localization in Rab27a-KD cells, which should have an accumulation of secretory MVE downstream of early endosomes due to the docking defect^22^.

To visualize exosome secretion in migrating cells, wide-field epifluorescence movies of HT1080 cells expressing pHLuorin-CD63 and mCherry-paxillin migrating in two-dimensinal were performed (Fig. 6b and Supplementary Movie 6). Overall, pHLuorin-CD63 fluorescence was observed at the front of the cell in thick ruffles and at the back of the cell at attachment sites. At the back of the cell, a striking feature was prominent ‘slime trails’ of fluorescence that were frequently left behind the migrating cells (Fig. 6b,c). Examination of phase-contrast images from the same movies reveals that the rear of the cell is far in advance of the ‘slime trails’ and does not reveal any apparent bits of torn-off plasma membrane (Fig. 6b). Furthermore, examination of mCherry-paxillin fluorescence gathered at the same time shows that only green fluorescence is present in the trail (Fig. 6b and Supplementary Movie 6). These data suggest that the primary source of the slime trail is exosomes and not torn-off plasma membrane or large adhesions left behind. These trails appeared to serve an adhesive function based on attachment to membrane at the rear of the cell that is particularly evident in the movies (Supplementary Movie 6). To further analyse the localization of pHLuorin-CD63 with respect to leading-edge protrusions, higher speed movies were taken. Kymographs showing protrusion at the leading edge over time were obtained and revealed that pHLuorin-CD63 was not enriched in the initial flat lamellipodial protrusions (Fig. 6c and Supplementary Movie 7). Instead there was an increased fluorescence in membranes that ruffled back, which could be due to membrane thickness or adhesion of exosomes after the initial membrane protrusion.

**Figure 7:**
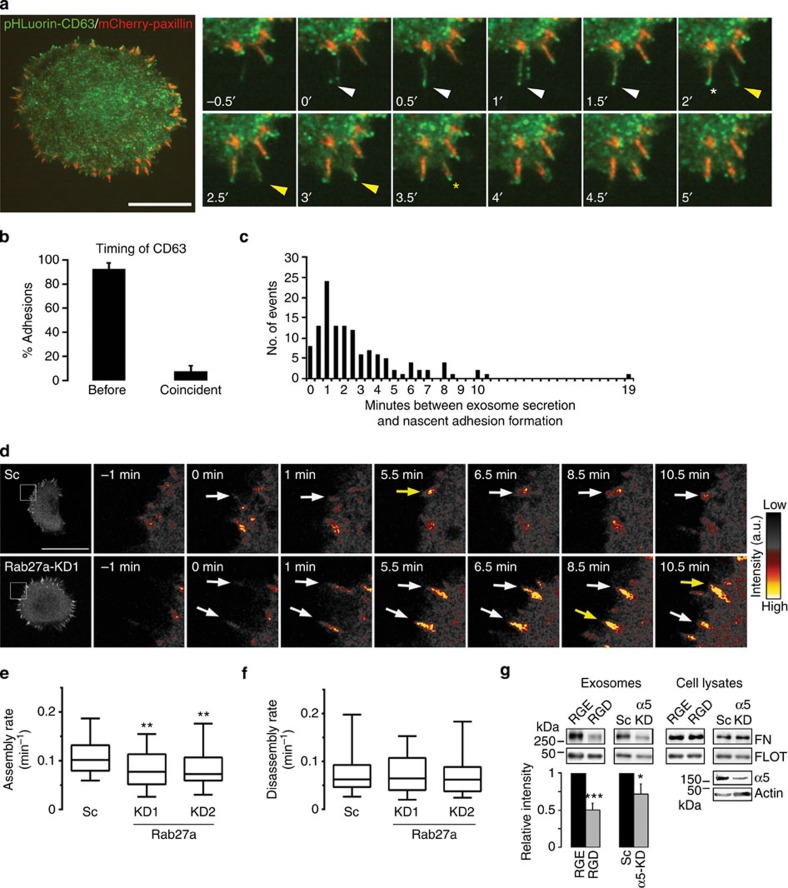
Exosomes promote adhesion assembly (**a**) Image from TIRF movie of pHLuorin-CD63 (green) and mCherry-paxillin (red)-expressing HT1080 cell on FN-coated (1 μg ml^−1^) MatTek dishes. Time series shows bursts of pHLuorin-CD63 fluorescence (arrowheads) preceding adhesion formation (indicated by *, red paxillin fluorescence visible at that time). Two adhesion formation events are, respectively, indicated with white and yellow markers. Representative of *n*=7 movies. See also Supplementary Movie 8. (**b**) Quantitation of per cent of adhesions with a pHLuorin-CD63 fluorescence burst before or coincident with their formation. (**c**) Quantitation of the number of minutes between pHLuorin-CD63 appearance and adhesion formation. (**d**) Representative time series of GFP-paxillin-marked adhesion assembly and disassembly for control (Sc) and Rab27a-KD HT1080 cells on FN-coated (1 μg ml^−1^) MatTek dishes. Magnifications of boxed area are shown at right by heat map coloration to show intensity. 0 min indicates start of adhesion formation. White arrows indicate example adhesions and yellow arrows indicate the peak adhesion intensity. Asterisk indicates a newly forming adhesion. Note that a longer period of time is required for adhesion assembly in KD cells. (**e**,**f**) Box-and-whisker plots of adhesion assembly (**e**) and disassembly (**f**) rates. Each box shows the 25–75th percentile and whiskers indicate the 5–95th percentile and the black line indicates the median. Analysis from >30 adhesions for **e** and >40 adhesions for **f** from three to eight cells per condition. See also Supplementary Movie 9. (**g**) Western blot analysis of FN association with exosomes. GRGESP (100 μM; RGE, control) or GRGDSP (100 μM; RGD, integrin-binding) peptides were added to cultured HT1080 control cells (Sc) for 48 h before exosome isolation. Equal exosome numbers were loaded on western blots. Quantitations show FN intensity normalized to flotillin intensity as a loading control from *n*≥4 western blots. α5-KD, integrin α5-knockdown-HT1080. *P<0.05; **P<0.01; ***P<0.001 compared with Sc using Mann–Whitney *U*-test. Scale bars in **a** and **d** represent 30 μm.

To further define the relationship between exosome secretion and adhesion formation, we performed live total internal reflected fluorescence (TIRF) microscopy of cells expressing both pHLuorin-CD63 and mCherry-paxillin (Fig. 7a and Supplementary Movie 8). To visualize newly forming adhesions, movies were taken in actively spreading cells. Interestingly, bursts of pHLuorin-CD63 closely preceded adhesion formation. Analysis of these data revealed that 93% of adhesions were preceded by a burst of CD63-pHLuorin, with a median of 2 min lag between CD63 appearance and adhesion formation (Fig. 7b,c).

To specifically test the role of exosome secretion in adhesion formation, we analysed the dynamics of adhesions in GFP-paxillin-expressing control and Rab27a-KD cells. Confocal movies taken in actively spreading cells revealed that Rab27a-KD cells have a selective defect in the rate of adhesion assembly (Fig. 7d,e and Supplementary Movie 9). There was no difference in the rate of adhesion disassembly (Fig. 7f) or in the overall number of peripheral adhesions per frame of the movies (25.16±4.37 for HT1080^Scrambled^, 27.65±3.08 (*P*=0.3229 compared with scrambled) for HT1080^Rab27aKD1^ and 30.40±4.24 (*P*=0.2112 compared with scrambled) for HT1080^Rab27aKD2^, *P* by Student’s *t*-test). Overall, the slow adhesion assembly time led to a significant prolongation of the overall adhesion lifetime (average lifetime *t*_1/2_ for HT1080^Scrambled^ adhesions=7.0±0.4 min, compared with HT1080^Rab27aKD1^=10.7±0.8 min (*P*<0.002, Student’s *t*-test) and HT1080^Rab27aKD2^=10.4±1.0 min (*P*<0.002, Student’s *t*-test). These data are consistent with the known relationship between dynamic adhesions and fast migration speeds^32^,^33^,^34^.

### FN is targeted to exosomes by interactions with integrins

Our data indicate that FN becomes specifically targeted to exosomes in a form that promotes efficient adhesion formation. Soluble FN becomes converted to a fibrillar, adhesion-promoting form in association with cellular integrin receptors^35^, which are also frequent exosome cargos. To test whether targeting of FN to secreted exosomes depends on binding to integrins, we inhibited FN–integrin interactions and assessed the resulting FN content of exosomes. Treatment of HT1080 cells with integrin-binding RGD peptide for 48 h greatly decreased the FN content of exosomes compared with cells treated with control RGE peptide (Fig. 7g). Likewise, inhibition of the FN-binding integrin α5β1 by KD of α5 also decreased the FN content of exosomes (Fig. 7g). These data indicate that FN is targeted to exosomes by binding to cognate cellular integrin receptors, and then secreted in adhesive form to promote adhesion formation and effective cell motility.

## Discussion

**Figure 8:**
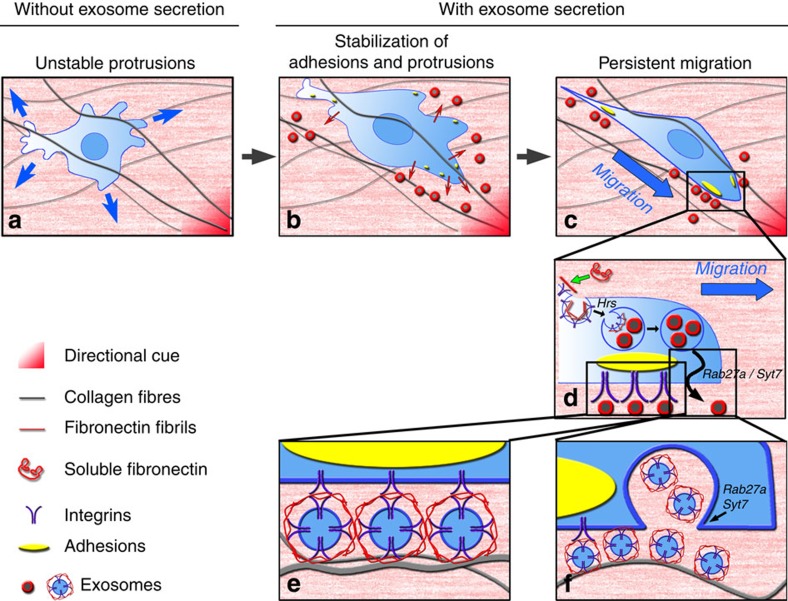
Proposed model for exosome control of directionally persistent *in vivo* cell migration (**a**) In the absence of exosome secretion, cells have unstable protrusions (blue arrows) and are unable to migrate effectively. (**b**) Secretion of exosomes (yellow) allows cells to effectively respond to directional cues by reinforcing nascent adhesions (orange) and stabilizing protrusions. (**c**) Positive feedback from exosomes promotes effective and directionally persistent motility, through adhesion enhancement and potentially additional unknown mechanisms. (**d**) ECM cargoes, such as FN, are carried on exosomes through a process involving endocytosis of ECM-integrin complexes, such as FN-α5β1, and subsequent sorting into MVEs. (**e**,**f**) Autocrine secretion of FN-coated exosomes at the leading edge may allow decoration of collagen fibrils with FN-bound exosomes that can then interact with cellular integrin receptors. Locally elevated concentration of FN-bound exosomes via secretion could facilitate integrin clustering and strong adhesion formation leading to accelerated migration. ECM carried on autocrine-secreted exosomes may be particularly effective at promoting cellular adhesion by matching matrix ligands to the adhesion receptor repertoire of the cell.

Cell movement through tissues is a complex process that requires a coherent response to potentially divergent tissue cues. Here we identify exosome secretion as a key autocrine mechanism to reinforce directional cues and support productive migration (see model in Fig. 8). *In vivo*, inhibition of exosome formation, MVE docking or MVE fusion led to similar defects in cell migration speed and persistence. *In vitro*, purified exosomes, but not shed microvesicles, were able to rescue speed defects of exosome-inhibited cells. Both *in vitro* and *in vivo*, secretion of exosomes stabilizes nascent protrusions, a phenotype associated with cellular adhesion^19^,^20^,^21^. *In vitro* live-imaging experiments further demonstrated that a key function of exosomes is to promote adhesion assembly. A critical exosome cargo that drives the adhesion and speed of migrating cells is ECM, as demonstrated by the reduction in speed supported by FN-depleted exosomes and full rescue of speed defects in exosome-inhibited cells by purified FN. Altogether, our results demonstrate that autocrine exosome secretion promotes both efficient and directional cell motility and indicates that a major function of ECM in exosomes is to promote adhesion formation and enhance cell speed.

While exosomes have been implicated in cell motility previously^14^,^15^,^26^, it has not been clear how essential to the process they are or what specific steps are regulated. Our data now demonstrate that autocrine secretion of exosomes controls both directional persistence and speed of cells migrating *in vivo.* To understand in depth the role of exosome-delivered ECM in cell motility, we carried out a variety of live-imaging experiments to both visualize the spatiotemporal relationship between exosome secretion and motility structure formation and demonstrate specific defects of exosome-inhibited cells in adhesion assembly and cell speed but not in protrusion formation. Altogether, these data reveal a much more important role for exosome secretion in adhesion formation than previously appreciated^36^,^37^, and particularly highlight the key role of autocrine exosome secretion in motility processes.

A key question raised by our findings is why does the cell need to place FN on the outside of an exosome to promote adhesion assembly? FN is initially secreted from Golgi-derived vesicles in a soluble non-adhesion-promoting form and then assembled into fibres through interaction with integrins on the cell surface^35^. These fibres may not be optimally positioned to promote migration in one direction and their assembly may not be dynamic enough to respond to fluctuating directional cues. By contrast, endocytosis and resecretion of FN–integrin complexes on exosomes likely facilitates dynamic placement of highly competent adhesive substrata that promote cellular motility. In a previous study, we found that resecretion of fluorescent FN at the bottom of cells occurred within 1 h of its addition to the culture medium and was rapidly incorporated into nascent streak-like adhesion structures^9^. Presumably this endocytosis–resecretion process is happening continuously so that at any given time there may be a pool of matrix-carrying exosomes in MVE that could be rapidly secreted and used for migration.

FN is known to be endocytosed in association with integrins and targeted to MVE^8^ (Fig. 7g). During the intraluminal vesiculation process that creates exosomes within the MVE, the FN would be placed on the outside of the exosome (Supplementary Fig. 2) bound to integrins in the correct topology to interact with both the outside of the cell and with other ECM such as the collagen fibres in the chick CAM (see model, Fig. 8). *In vitro*, the major source of FN is serum. In the tumour microenvironment, the potential sources of soluble FN are many, including fibroblasts, endothelial cells and tumour cells, as well as leakage from the vasculature. It is also likely that additional matrix molecules could play a similar role, depending on the receptor profile of the migrating cells. In addition, matrix proteolysis could liberate adhesive fragments that could be internalized and then recycled on the outside of exosomes to promote dynamic adhesion formation and motility.

In addition to ECM, exosomes contain numerous cargoes that could impact cell motility in diverse contexts, including proteinases, growth, chemotactic and other extracellular factors^23^,^26^,^27^,^38^. Indeed, our *in vivo* data indicate that exosomes not only regulate cell speed but also directionality. Because Rab27a-KD cells did not have a defect in persistence in our random motility *in vitro* assays, we were not able to address exosome control of directionality in that experimental set-up. One possibility is that proteinases could facilitate persistent movement through tissues by remodelling impeding ECM. Another possibility is that exosomes mediate directional movement via chemokines or other factors. Specific identification of which exosome cargoes affect distinct aspects of cell motility is an important future area of research for both the exosome and motility fields. In addition, juxtaposition of multiple motility-promoting factors in a single exosome or closely apposed exosomes could synergistically modulate complex motility behaviours. Understanding these multivariate interactions will be an ongoing challenge for the field.

Use of the pHLuorin-CD63 probe to observe only extracellular CD63 allowed us to achieve a strong signal-to-noise ratio and observe dynamic relationships between exosome secretion and cellular structures. Our observation of adhesive trails left behind migrating cells is reminiscent of slime trails left by lower organisms, including snails, slugs and slime moulds^39^,^40^,^41^. Such slime trails contain complex mixtures of components and serve a variety of purposes, including providing adhesive traction without gluing the organism in place^39^,^40^, and, in the case of slime moulds, as a chemorepellant to prevent recrossing of the same track^41^. We speculate that trails of exosomes from mammalian cells may provide a similar mixture of functions, including not only adhesion but also the ability to modulate other cellular behaviours via diverse cargo molecules.

In this study, we identified a specific cellular organelle, the MVE, as critical to directional cell motility. As polarization is an intrinsic part of directional movement, this finding is consistent with recent findings that targeted MVE secretion is important for formation of the posterior pole of polarized Dictyostelia^42^. In addition, a recent study found that fibroblast-induced exosomes could mobilize Wnt11 from breast cancer cells to induce planar cell polarity signalling^27^. Interestingly, several molecules that have been previously shown to promote directional cell motility are sorted into exosomes and/or regulate their cargo content. Thus, syndecans were recently shown to control exosome biogenesis^43^ and also separately to control directional migration^1^. Likewise, Wnt molecules are major components of exosomes^44^ and together with syndecan-4 regulate directional cell motility *in vivo*^1^,^27^,^45^. On the basis of these studies and our findings, we speculate that a number of molecules known to control directional cell motility may be doing so via control of exosome biogenesis or cargo content, or as biological cargoes of exosomes.

In summary, we have shown that exosome secretion is essential for directional and efficient cell migration and enhances migration speed by delivering ECM to promote adhesion formation. These data identify a novel feedback pathway that enhances cell motility via local autocrine secretion.

## Methods

### Cell culture and reagents

HT1080 and GFP-expressing HT1080 cells^18^ were maintained in DMEM supplemented with 10% bovine growth serum (BGS). GFP-expressing HEp3 cells^18^ were maintained in DMEM supplemented with 10% fetal bovine serum. A lentiviral shRNA expression system, pLKO.1, was used to knockdown Syt7 (TRCN0000007967 (5′-CCGG-CCCTGAATGTCGAGGATAGTA-CTCGAG-TACTATCCTCGACATTCAGGG-TTTTT-3′ and TRCN0000007968 (5′-CCGG-GACGATGAAGAGGAACCTGAA-CTCGAG-TTCAGGTTCCTCTTCATCGTC-TTTTT-3′), Thermo Scientific), Rab27a (TRCN0000005296 (5′-CCGG-CGGATCAGTTAAGTGAAGAAA-CTCGAG-TTTCTTCACTTAACTGATCCG-TTTTT-3′) and TRCN0000005297 (5′-CCGG-GCTGCCAATGGGACAAACATA-CTCGAG-TATGTTTGTCCCATTGGCAGC-TTTTT-3′), Thermo Scientific), integrin α5 3′ UTR (TRCN0000230126 (5′-CCGG-AGGCAGATCCAGGACTATATT-CTCGAG-AATATAGTCCTGGATCTGCCT-TTTTTG-3′), Sigma-Aldrich) or scrambled control (Plasmid #26701 (5′-CCTAAGGTTAAGTCGCCCTCG-CTCGAG-CGAGGGCGACTTAACCTTAGG-3′), Addgene). Hrs shRNAs (5′-GACCTGCTGAAGAGACAAGTC-3′ and 5′-GCATGAAGAGTAACCACAGC-3′) were cloned into pLenti6/Block iT^23^. GFP-paxillin in pRS vector^32^ and mCherry-CD63 in pLenti6 (ref. 23) were used to make stably expressing cell lines. Low-expressing cells were sorted by fluorescence-activated cell sorting and used for experiments. mCherry-paxillin was a gift from Donna Webb (Vanderbilt University). pHLuorin-CD63 was generated by PCR resulting in the insertion of pHLuorin (a gift from Dr Philippe Chavrier, Institut Curie) between amino acids 42 and 43 in extracellular domain 1 of human CD63 cDNA (a gift from Dr Gillian Griffiths, Cambridge Institute for Medical Research) and recombined into pcDNA3.1. mCherry-Rab5a Q79L was from Addgene. DyLight 550 (62262, Thermo Scientific) was used to label human plasma FN (33016-015, Life Technologies) according to the manufacturer’s instructions. GRGESP (44-0-26) and GRGDSP (44-0-24A) peptides were purchased from American Peptide Company. Primary antibodies were: Anti-Syt7 (105 173, Synaptic System, 1:200), anti-Rab27 (R4655, Sigma, 1:1,000), anti-FN (610077, BD Biosciences, 1:5,000), anti-flotillin (610820, BD Biosciences, 1:1,000), anti-Hrs (M-79, Santa Cruz Biotechnology, 1:500), anti-integrin α5 (AB1928, Millipore, 1:1,000), anti-Hsp70 (sc-24, Santa Cruz Biotechnology, 1:2,000), anti-CD63 (ab68418, Abcam, 1:500) and anti-β-actin (Ac-74, Sigma, 1:5,000). HRP-, Alexa Fluor 680- or IRDye800- secondary antibodies (1:10,000) were from Santa Cruz Biotechnology, Invitrogen or Rockland, respectively. Blots were imaged and analysed using a ChemiDoc MP (Bio-Rad) or an Odyssey Infrared Imaging System (Li-Cor Biosciences). Images were cropped for presentation and uncropped blots are presented in Supplementary Fig. 6.

### The CAM model

Use of the chick CAM to model the formation of metastatic colonies and primary tumours is described in detail elsewhere^17^,^18^. In brief, fertilized eggs decanted into sterilized weigh boats were allowed to mature ex ovo for 9 days. GFP-expressing cells were injected either i.v. or within the CAM tissue on developmental day 10. At indicated times, the ex ovo chick embryos were placed in a custom intravital imaging chamber^46^,^47^. Images of tumour cells in the CAM were acquired using a Lumar V12 fluorescence stereomicroscope (Zeiss) or an Olympus BX61 equipped with a digital camera controlled with Volocity image acquisition software (PerkinElmer).

### *In vivo* analysis of cell migration and protrusion dynamics

GFP-expressing HT1080 cells (1 × 10^5^) were injected i.v. into the CAM of ex ovo chicks on developmental day 10. At 24 h post injection, the ex ovo chicks were transferred to an intravital imaging chamber and placed under a fully automated temperature-controlled microscope (99 °F, BX61, Olympus^18^). Time-lapse migration movies were captured every 15 min for 16 h using × 10/0.40 UPlanSApo objective lens, whereas protrusion dynamics movies were captured at every 2 min for 1 h using × 20/0.70 W UApo/340 water objective lens. To analyse *in vivo* cell migration, the nuclear position of each cell was tracked and cell speed, displacement rate and persistence index were analysed using Volocity. For protrusion dynamics analysis, the aspect ratio was calculated using Fiji, the number of protrusions formed was counted manually and length and persistence of protrusions were measured using Metamorph software (Molecular Devices).

### Isolation of exosomes

To collect conditioned media, 80% confluent cells were cultured for 48 h in Opti-MEM. Exosomes were isolated from conditioned media by serial centrifugation at 300*g* for 10 min, 2,000*g* (4,000 r.p.m. in Ti45 rotor) for 30 min, 10000*g* for 30 min (9,300 r.p.m. in Ti45) and 100,000*g* (30,000 r.p.m. in Ti45) for overnight to, respectively, sediment live cells, dead cells, debris and microvesicles and exosomes (∼3.5 μg exosomes from 1 × 10^6^ HT1080s). For further purification of UC-exosomes, the discontinuous iodixanol gradient was prepared^48^. Solutions (40% (w/v), 20% (w/v), 10% (w/v) and 5% (w/v)) of iodixanol were made by diluting OptiPrep (60% (w/v) aqueous iodixanol, Axis-Shield PoC) with 0.25 M sucrose/10 mM Tris, pH 7.5 from the bottom to the top of a 14 × 89 mm polyallomer tube. UC-exosomes were added on a top of the gradient and continuous gradient was made through UC at 100,000*g* (24,000 r.p.m. in SW40 Ti rotor) for 18 h. Twelve fractions were collected and each fraction was diluted in PBS. Each fraction was pelleted through another round of ultracentrifugation at 100,000*g* for 3 h, washed with PBS and resuspended in PBS. To deplete FN in exosomes, cells were cultured in DMEM supplemented with 10% FN-depleted BGS^9^ for 10 days before collecting conditioned medium for exosome isolation. Exosomes were quantitated by NanoSight tracking analysis.

### *In vitro* single-cell migration assay

For coating experiments, tissue culture 24-well plates were incubated with appropriately exosome or human plasma FN (Gibco BRL) suspensions in PBS at 4 °C overnight before washing and using. Trypsinized cells (3,000 in each well) were then cultured overnight on the coated plates before capturing and analysing time-lapse migration movies using a Nikon Eclipse TE2000E microscope equipped with a 37 °C chamber as previously described^9^. The nuclear position of each cell was tracked and cell speed and persistence index was calculated using Metamorph software. For *in vitro* migration with add-back of exosomes in the media, cells were allowed to adhere to tissue culture well plates for 5 h before changing the media to DMEM supplemented with 10% exosome-depleted BGS (prepared by ultracentrifugation for 12 h at 100,000*g*)± purified exosomes. Then, live-cell imaging was performed as described above.

### Adhesion assembly and disassembly assay

GFP-paxillin-expressing cells were plated on glass-bottom dishes (MatTek) coated with 1 μg ml^−1^ FN and allowed to adhere for 1 h. Images were acquired with an LSM 510 laser scanning confocal microscope (CarlZeiss) equipped with a × 63/1.40 NA Plan Apo oil objective lens at 37 °C with 5% CO_2_. Images were taken every 30 s for 45 min. The rate constants for assembly and disassembly of adhesions were measured through the Focal Adhesion Analysis Server^49^.

### Live imaging of CD63 and focal adhesions

Either mCherry-CD63 was stably expressed in stable GFP-paxillin-expressing HT1080 or pHLuorin-CD63 was transiently co-transfected into HT1080 along with mCherry-paxillin or mCherry-Rab5A Q79L. A day after transfection, cells were replated on glass-bottom dishes coated with FN (1 μg ml^−1^). Images of mCherry-CD63/GFP-paxillin- or mCherry-Rab5A Q79L/pHLuorin-CD63-expressing cells were acquired with LSM 710 META laser scanning confocal microscope equipped with a × 63/1.40 Plan-Apochromat oil objective lens at 37 °C with 5% CO_2_. Epifluorescence images of pHLuorin-CD63-expressing cells were acquired with Nikon Eclipse TE2000E microscope equipped with a 37 °C chamber using a × 40/0.60 Plan Fluor lens. For migration movies, images were taken every 2 min for 4 h. For lamellipodial protrusion analysis by kymography, images were taken every 6 s for 20 min. Images of pHLuorin-CD63/mCherry-paxillin-expressing cells were taken with a Nikon Eclipse TiE TIRF microscope equipped with a Perfect Focus System using a TIRF × 100/1.49 NA oil-immersion lens and Neo 5.5 cMOS camera (Andor). For each cell, images were taken every 30 s for 45 min, yielding 91 frames per movie.

### Numbers and statistics

For both quantitated data and representative images from experiments, the *n* values and independent experiment numbers are listed in the figure legends. Imaging data were acquired from at least three independent experiments and multiple images or movies on multiple cells were captured. Cell numbers to be quantitated for motility experiments were determined by our experience and analyses were excluded only if there is an obvious reason for poor data, such as dead or sick-looking cells. For non-quantitated western blots (for example, checking knockdown), they were generally performed a single time. All data sets were tested for normality using the Kolmogorov–Smirnov normality test in SPSS software (IBM). Non-parametric data groups were compared using the Mann–Whitney *U*-test and plotted as box and whiskers, in which the box indicates the 25–75th percentile and the whiskers indicate the 5–95th percentile, with the line indicating the median. Parametric data were compared using Student’s *t*-test and plotted as mean±s.e. in bar graphs.

## Additional information

**How to cite this article:** Sung, B.H. *et al.* Directional cell movement through tissues is controlled by exosome secretion. *Nat. Commun.* 6:7164 doi: 10.1038/ncomms8164 (2015).

## Supplementary Material

Supplementary Figures and References Supplementary Figures 1-6 and Supplementary References

Supplementary Movie 1 In vivo migration of HT1080-Scrambled (Sc) control, Synaptotagmin7 (Syt7)- knockdown (KD), and Rab27a-knockdown cells within the CAM. Representative migration for each cell line is shown in overlay lines. Time-lapse images were taken every 15 min for 16 h. Note the lack of directionally persistent migration in Syt7- and Rab27a-KD cells.

Supplementary Movie 2 In vivo migration of HT1080-Scrambled (Sc) control, Synaptotagmin7 (Syt7)- knockdown (KD), and Rab27a-knockdown cells within the CAM. Representative migration for each cell line is shown in overlay lines. Time-lapse images were taken every 15 min for 16 h. Note the lack of directionally persistent migration in Syt7- and Rab27a-KD cells.

Supplementary Movie 3 Protrusion dynamics of HT1080-Scrambled (Sc) control, Synaptotagmin7 (Syt7)-knockdown (KD), and Rab27a-knockdown cells within the CAM. High resolution in vivo images were taken every 2 min for 1 h to compare protrusion dynamics. Note the unpolarized, protrusive phenotype of KD cells.

Supplementary Movie 4 3D projection of DyLight550-FN localization both within and outside of an HT1080 cell in the chick CAM. Note incorporation of the FN into adhesions associated with cellular protrusions.

Supplementary Movie 5 Trafficking of mCherry-CD63-positive vesicles (red) to adhesions labeled with GFP-paxillin (green). Arrows indicate colocalization of mCherry-CD63 with adhesions whereas arrowhead indicates localization of paxillin within endosomes. Time-lapse images were taken every 7 sec for 30 frames.

Supplementary Movie 6 Live epifluorescence imaging of pHLuorin-CD63 (green) and mCherry-paxillin (red) in migrating cells reveals adhesive exosome trails left behind the cell. Time-lapse images were taken every 2 min for 4 h.

Supplementary Movie 7 Fast epifluorescence imaging of protrusions in pHLuorin-CD63 reveals no enrichment of CD63 in flat initial protrusions, only in ruffles. Time-lapse images were taken every 6 sec for 20 min. Compare to kymograph in Fig 5c'.

Supplementary Movie 8 Live TIRF imaging of pHLuorin-CD63 (green) and mCherry-paxillin (red) reveals bursts of CD63 fluorescence preceding visible adhesion formation. Magnification of boxed area is shown at right. Time-lapse images were taken every 30 sec for 45 min.

Supplementary Movie 9 Heatmap-colored live confocal imaging for focal adhesion dynamics in scrambled control (Sc) and Rab27a-knockdown (KD) cells expressing GFP-paxillin. Time-lapse images were taken every 30 sec for 45 min. Yellow arrows indicate the peak of fluorescence intensity, which marks the end of the adhesion assembly time. Note the slower assembly time for adhesions in Rab27a-KD cells compared to control cells.
