# Supplementary material for: Directional cell movement through tissues is controlled by exosome secretion
Source: Nat Commun. 2015 May 13;6:7164. doi: 10.1038/ncomms8164 (PMC4435734; doi:10.1038/ncomms8164)
Supplement: Supplementary Figures and References — Supplementary Figures 1-6 and Supplementary References [file ncomms8164-s1.pdf]

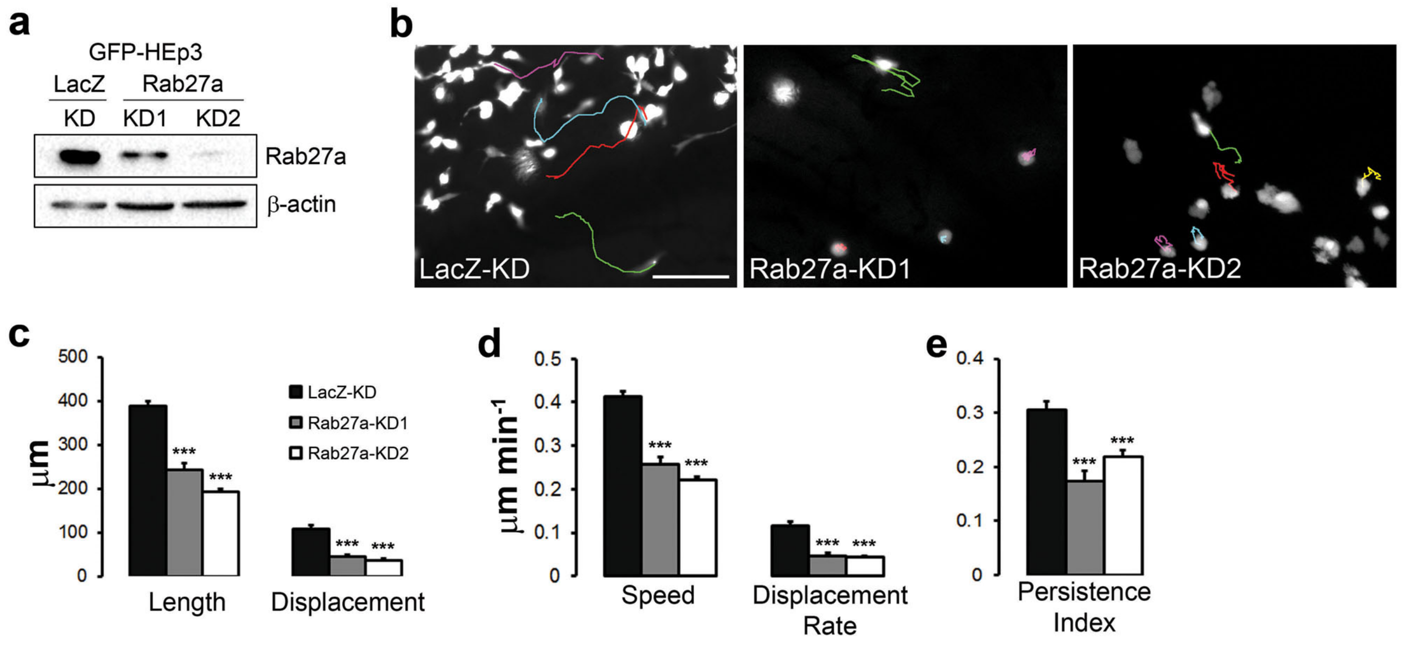

Supplementary Figure 1. Rab27a-KD inhibits speed and persistence of HEp3 cells migrating in the chick CAM. (**a**) Western blot analysis of Rab27a expression in GFP-expressing HEp3 cells. (**b**) Representative images of HEp3 cells migrating within the CAM. Representative cell tracks are shown. Scale bar = 200 μm. (**c**) Length and Displacement plots. (**d**) Speed, calculated by Length divided by time. Displacement Rate, calculated by Displacement divided by time. (**e**) Persistence index, calculated as Displacement Rate divided by Speed. Error bars = standard error of the mean (SEM) from 3 independent experiments (n > 45 cells for each cell line). *** $p < 0.001$ compared to Sc.

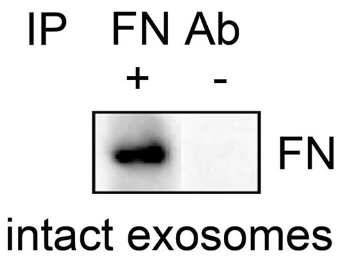

Supplementary Figure 2. Fibronectin is present at the surface of exosomes. To determine whether fibronectin (FN) is present on the outside surface of exosomes, intact exosomes were subjected to immunoprecipitation with anti-fibronectin antibody (+) or horse serum (-) and then analyzed by Western blot analysis for FN.

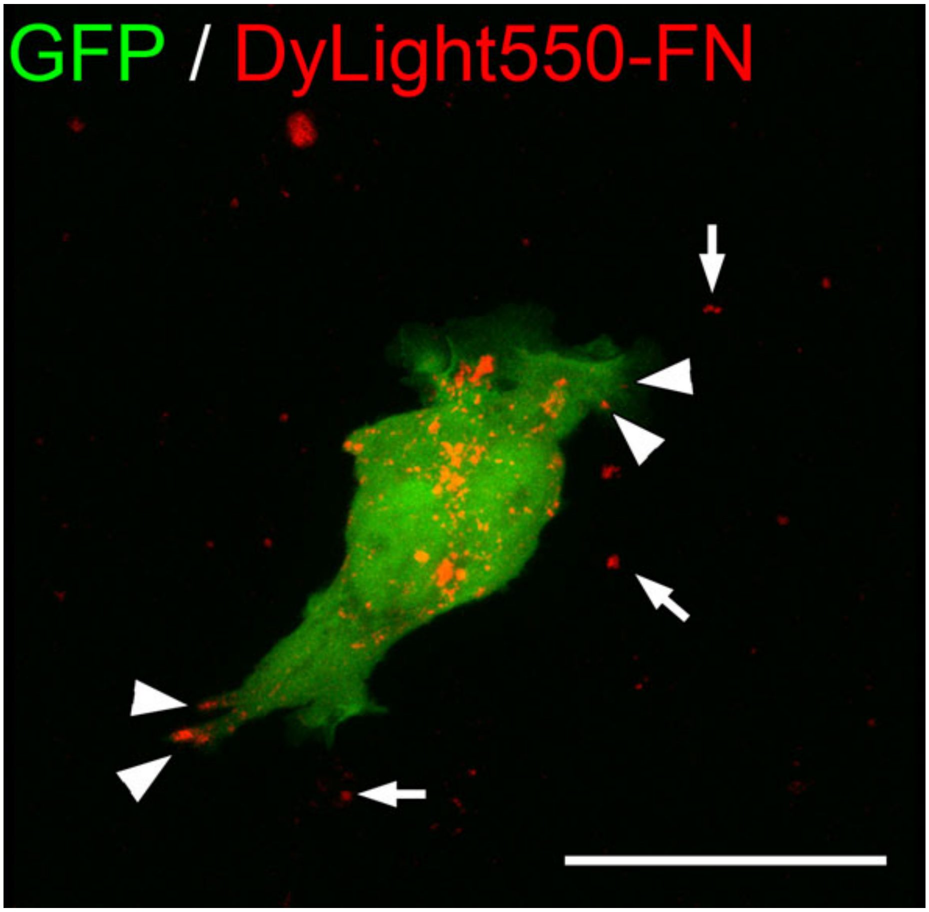

Supplementary Figure 3. Incorporation of endocytosed FN into adhesions *in vivo*. DyLight550-labeled FN (red) was allowed to be endocytosed into GFP-expressing HT1080 (green) for 1 h. DyLight550-FN-loaded HT1080 cells were then trypsinized and injected IV within the chick CAM. After 24 h, the chick CAM was observed by confocal microscopy (LSM510). Arrowheads indicate incorporation of endocytosed FN into adhesions associated with apparent leading edge protrusions and rear cell edge. Arrows indicate resecreted FN present in the chick CAM. Scale bar = 30 μm. Representative of 9 movies.

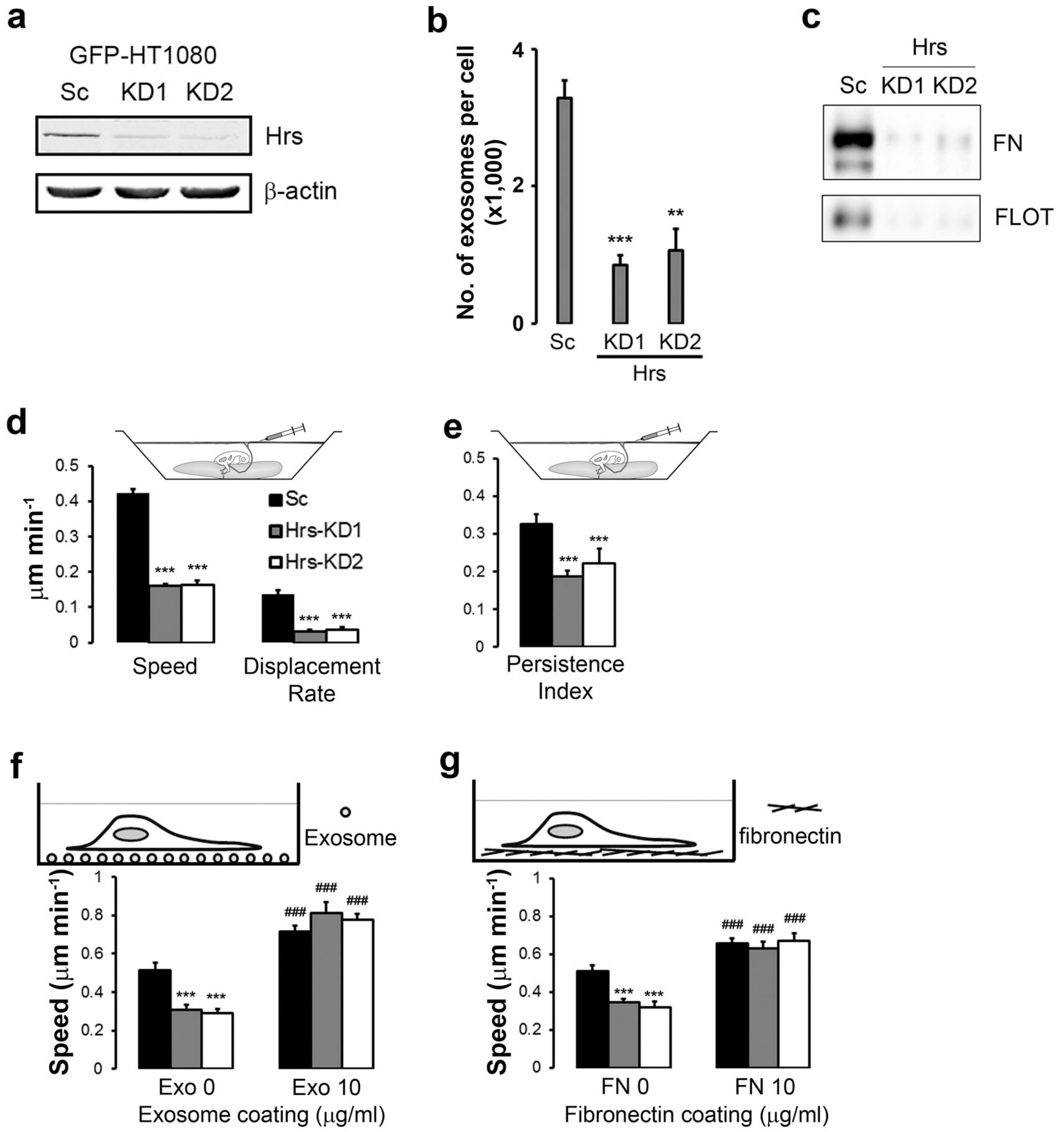

Supplementary Figure 4. Exosome biogenesis is critical for persistent and fast migration. (**a**) Western blot analysis of Hrs expression in GFP-expressing HT1080 cells. (**b**) Average number of exosomes secreted from Hrs-KD cell lines from 3 independent experiments. (**c**) Western blots of exosome cargos. FN, fibronectin; FLOT, flotillin. Sc, scrambled shRNA control; KD, Hrs-knockdown. (**d**) Speed and displacement rate, and (**e**) Persistence index of control and Hrs-KD cell lines in the chick CAM (*in vivo* migration). n > 40 cells from three independent experiments for each cell line. (**f and g**) Single cell *in vitro* migration on exosome (Exo)-coated (**f**) or FN-coated (**g**) plates. n ≥ 18 cells for each cell line from 3 independent experiments. Error bars = SEM. ** $p < 0.01$; *** $p < 0.001$ compared to Sc. ### $p < 0.001$ compared to same cell line on Exo 0 or FN 0.

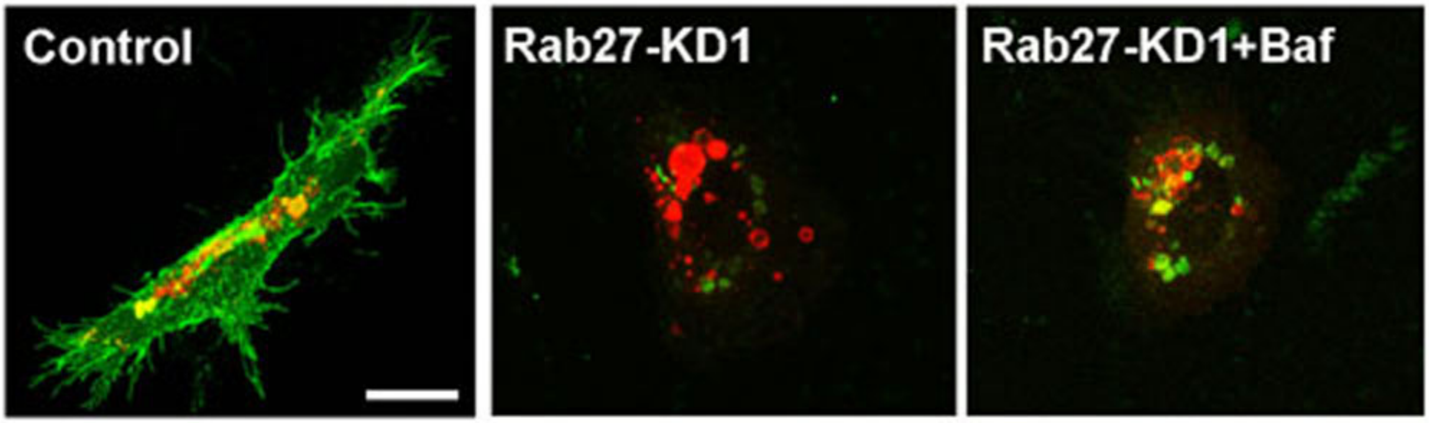

Supplementary Figure 5. Validation of pHLuorin-CD63 construct. pHLuorin-GFP is a pH-sensitive GFP with a pKa of 7.1 [1]. Therefore, the fluorescence is quenched at low pH, such as that found in late endosomes, but bright at neutral pH, such as the extracellular space and early endosomes. In contrast to control cells, pHLuorin-CD63 fluorescence (green) is not found on the cell surface in Rab27a-KD cells, consistent with lack of exosome secretion in those cells. Red=mCherry-Rab5a Q79L, which both marks and leads to enlargement of early-to-late transitioning endosomes, which will not be fully acidified yet. Note the presence of pHLuorin-CD63 in acidic late endosomes in Rab27a-KD cells revealed by Bafilomycin treatment (+Baf) for 75 min to induce neutral pH of endosomes. Note that in control cells much of the internal pHLuorin-CD63 colocalizes with Rab5a Q79L endosomes, which will have higher pH than later endosomal compartments. In Rab27a-KD cells, there is less colocalization even in the presence of Bafilomycin, presumably because the docking defect of Rab27a-KD cells [2] leads to accumulation of late endosomes downstream of Rab5 (evident in Bafilomycin-treated cells). Scale bar = 20 μm. Representative of 3 movies for control cells and 5 movies for Rab27a-KD cells.

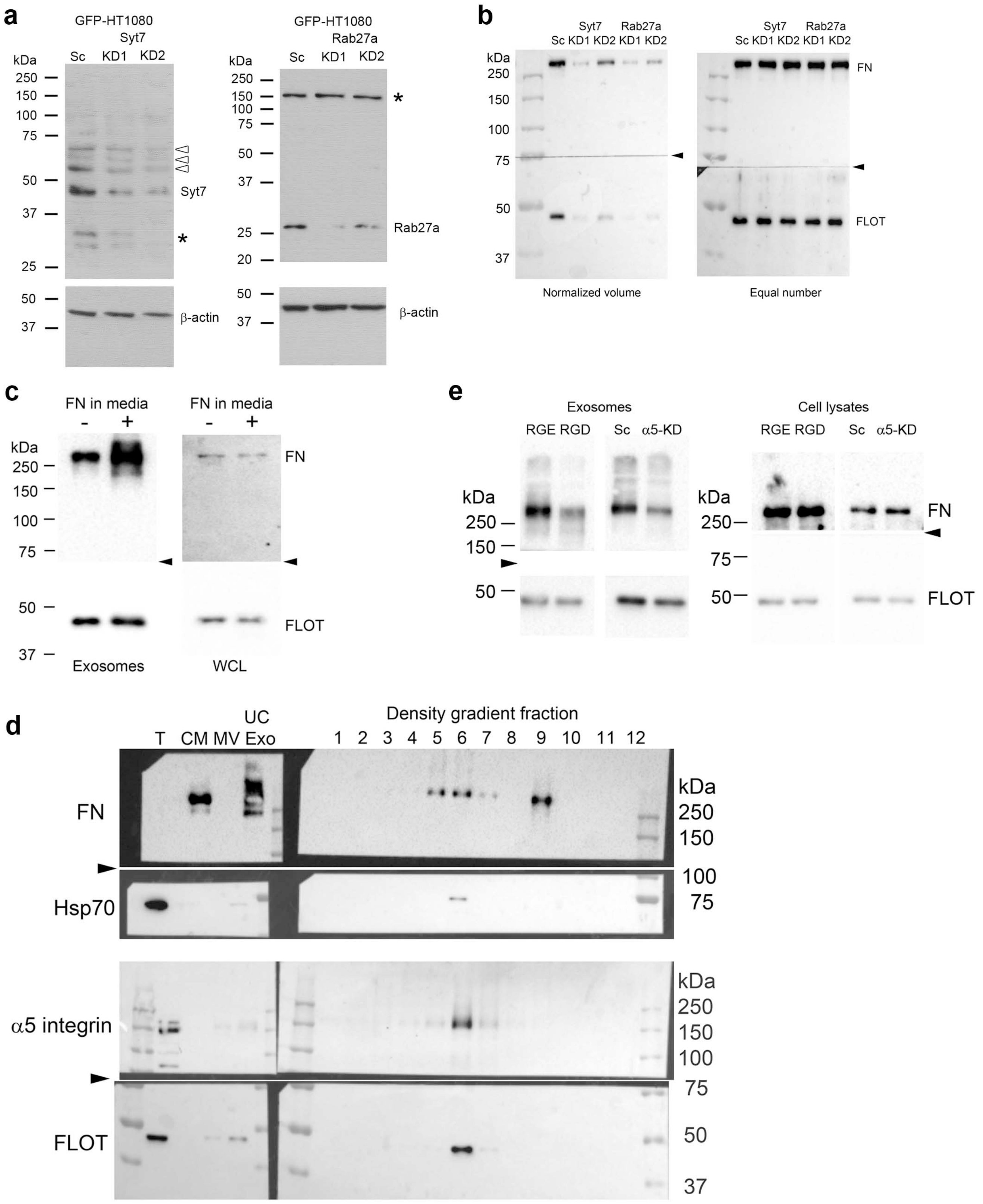

Supplementary Figure 6. Uncropped Western blots. Uncropped blots for Fig 1a **(a)**, 4b **(b)**, 4g **(c)**, 5b **(d)**, and 7g **(e)**. **(a)** Blots were probed with synaptotagmin 7 (Syt7) or Rab27a antibodies and reprobed with a β-actin antibody. Empty arrowheads indicate isoforms. Non-specific bands are marked by asterisks. Cut strips were probed with FN and flotillin (FLOT) antibodies **(b, c,** and **e)**. **(d)** Cut strips were probed with FN and Hsp70 antibodies or probed by α5 integrin and FLOT antibodies. Black arrowheads indicate where the blots were cut.

Supplemental References

1. Sankaranarayanan S, De Angelis D, Rothman JE, Ryan TA. The use of pHluorins for optical measurements of presynaptic activity. *Biophys J* **79**, 2199-2208 (2000).

2. Ostrowski M*, et al.* Rab27a and Rab27b control different steps of the exosome secretion pathway. *Nat Cell Biol* **12**, 19-30; sup pp 11-13 (2010).
